# Consumption of ultra-processed foods and its association with sociodemographic factors in the adult population of the 27 Brazilian state capitals (2019)

**DOI:** 10.11606/s1518-8787.2021055002833

**Published:** 2021-07-27

**Authors:** Caroline dos Santos Costa, Isabela Fleury Sattamini, Eurídice Martinez Steele, Maria Laura da Costa Louzada, Rafael Moreira Claro, Carlos Augusto Monteiro

**Affiliations:** I Universidade de São Paulo Faculdade de Saúde Pública Programa de Pós-Graduação em Nutrição em Saúde Pública São PauloSP Brasil Universidade de São Paulo. Faculdade de Saúde Pública. Programa de Pós-Graduação em Nutrição em Saúde Pública. São Paulo, SP, Brasil; II Universidade de São Paulo Núcleo de Pesquisas Epidemiológicas em Nutrição e Saúde São PauloSP Brasil Universidade de São Paulo. Núcleo de Pesquisas Epidemiológicas em Nutrição e Saúde. São Paulo, SP, Brasil; III Universidade de São Paulo Faculdade de Saúde Pública Departamento de Nutrição São PauloSP Brasil Universidade de São Paulo. Faculdade de Saúde Pública. Departamento de Nutrição. São Paulo, SP, Brasil; IV Universidade Federal de Minas Gerais Escola de Enfermagem Departamento de Nutrição Belo HorizonteMG Brasil Universidade Federal de Minas Gerais. Escola de Enfermagem. Departamento de Nutrição. Belo Horizonte, MG, Brasil

**Keywords:** Diet, Adults, Ultra-processed Food, Sociodemographic Distribution, Brazil

## Abstract

**OBJECTIVE:**

To describe the magnitude of consumption of ultra-processed foods in the adult population (≥ 18 years old) in the capitals of the 27 federative units of Brazil, as well as its association with sociodemographic variables.

**METHODS:**

Data used in this study stem from participants (n = 52,443) of the 2019 wave of the annual survey of the “National surveillance system for risk and protective factors for chronic diseases by telephone survey” (Vigitel). The consumption of ultra-processed foods was described based on a score, corresponding to the sum of positive responses to questions about consumption on the previous day of thirteen subgroups of ultra-processed foods frequently consumed in Brazil. Poisson regression models were used to describe the crude and adjusted associations between high consumption of ultra-processed foods (scores ≥ 5) and sex, age group, and level of education.

**RESULTS:**

The frequency of high consumption of ultra-processed foods was 18.2% (95% CI 17.4–19.0). With or without adjustment for other sociodemographic variables, this frequency was significantly lower in females and decreased linearly with age. In the crude analysis, there was an increase in the frequency of high consumption from the lower level to the intermediate level of education and a decrease in this consumption from the intermediate level to the upper level. In the analysis adjusted for sex and age, the frequency of high consumption of ultra-processed foods was significantly lower at the higher level of education (12 or more years of study), with no differences between the other levels.

**CONCLUSION:**

Ultra-processed foods are consumed with high frequency in the adult Brazilian population in the 27 capitals of the federation. Being male, younger and having less education than university are conditions that increase, independently, the consumption of these foods.

## INTRODUCTION

Ultra-processed foods are industrial formulations of substances derived from foods that contain little or no whole food and are often added with flavorings, dyes, emulsifiers and other additives with a cosmetic function ^1,2^. Time series on the sale of ultra-processed foods in 80 countries indicate substantial annual increases in the global consumption of these products, especially in countries in less economically developed regions ^3^.

In Brazil, the sale of ultra-processed foods corresponded to 448 kcal per capita/day in 2014, a value 10.4% higher than that recorded in 2009^4^. National food purchasing surveys show that the contribution of ultra-processed foods to the total calories purchased by Brazilian households increased from 12.6% in 2002–2003 to 16.0% in 2008–2009 and to 18.4% in 2017–2018^5^. In 2017–2018, ultra-processed foods represented about 20% of the total calories consumed by adolescents and adults in Brazil^5^.

Population-based studies of several countries, including Brazil, show that the consumption of ultra-processed foods is associated with diets with higher energy density, with more sugar and unhealthy fats and with a lower content of fiber, protein, vitamins and minerals and, therefore, to diets that increase the risk of chronic noncommunicable diseases (NCDs)^6^. Systematic reviews of the literature on cohort studies conducted with adult populations in Brazil and other countries show that the consumption of ultra-processed foods is effectively associated with a higher incidence of NCDs, including obesity, high blood pressure, diabetes, cardiovascular diseases, cancer and depression^12,13^. Three cohort studies^14^ identified associations between consumption of ultra-processed foods and all-cause mortality and a randomized *cross-over* clinical trial showed that ultra-processed diets determine a daily increase of about 500 kcal in food consumption and, in two weeks, a accumulated difference of about 2 kg in body weight^17^.

Due to evidence that links ultra-processed foods to health and the upward trend of participation of these foods in the Brazilian diet, one of the central recommendations of the food guide of the Ministry of Health of Brazil is to avoid the consumption of ultra-processed foods^18^.

Since 2006, Brazil has had a national system for annual monitoring of risk and protective factors for chronic non-communicable diseases. This system, called Vigitel (“National surveillance system for risk and protective factors for chronic diseases by telephone survey”), was developed and tested in the capital of the State of São Paulo between 2003 and 2005^19,20^. Vigitel operates through telephone interviews carried out on probabilistic samples of the adult population with a landline and resident in the capitals of all 27 states. Using sample weights, the system provides annual estimates of the frequency and distribution of risk or protective factors for chronic diseases for the adult population residing in each capital and in the group of capitals^21^. For example, based on the Vigitel system, it is estimated that, between 2006 and 2019, the prevalence of obesity in the adult population in the group of 27 Brazilian capitals increased from 11.8% to 20.3%, while the prevalence of smokers decreased from 15.7% to 9.8%.

In 2019, the annual telephone survey of the Vigitel system included a module of questions on the consumption of ultra-processed foods. Based on this module, this study aimed to estimate the consumption of ultra-processed foods in the adult population living in the capitals of the 27 federative units in Brazil and to investigate its association with sociodemographic variables.

## METHODS

All data used in this study come from the 2019 wave of the annual cross-sectional survey of the Vigitel system^21^.

### Sampling

In 2019, the Vigitel system interviewed 52,443 people aged 18 years or over living in the capitals of Brazil’s 27 federative units.

The sampling process used by Vigitel is described in detail in the annual reports of the system^21^. In summary, based on registrations by fixed-line operators, 5,000 fixed telephone lines are drawn by capital by systematic drawing and stratified by postal address code (CEP). These lines are then grouped into replicas of 200 lines, each replica reproducing the same proportion of lines per zip code as the original record. Eligible lines are those corresponding to households where there is at least one resident 18 years of age or older. For each eligible line, one of the adult residents is drawn and interviewed. Calls are made to the lines contained in each replica successively and until a total of approximately two thousand interviews are reached in each capital.

### Data Collection

Vigitel system interviews are conducted over the twelve months of the year through telephone calls made by trained operators. The system questionnaire includes questions with a brief statement and pre-coded response categories on sociodemographic characteristics, consumption of tobacco and alcoholic beverages, diet and physical activity, among others^21^. In 2019, the Vigitel questionnaire included a module of questions on the consumption on the previous day (yes or no) of 13 subgroups of ultra-processed foods, selected from those most consumed in Brazil according to the national food consumption survey carried out as part of the Household Budget Survey (POF) of the IBGE 2008–2009^6^. They are: soft drinks; fruit juice in box or can; powdered soft drink; chocolate drink; flavored yogurt; packaged salty snacks or crackers; sweet cookie, stuffed cookie or packaged cake; chocolate, ice cream, gelatin, *flan* or other industrialized dessert; sausage, mortadella or ham; loaf, hot dog or hamburger bread; mayonnaise, ketchup or mustard; margarine; instant noodles, instant powdered soup, frozen lasagna or other ready-to-eat frozen dish.

### Data Analysis

Initially, we described the frequency of consumption on the day before the interview of each of the 13 subgroups of ultra-processed foods included in the questionnaire of the Vigitel system for the adult population of the 27 capitals. Then, we calculated for each individual an ultra-processed food consumption score that corresponds to the sum of positive responses to questions about the consumption of each of the subgroups of ultra-processed foods, which can therefore vary between zero and 13. Score distribution was presented graphically.

Estimates of the frequency of high consumption of ultra-processed foods (outcome, defined by scores ≥ 5) were presented for the entire adult population of the 27 capitals and according to sociodemographic variables (exposures), which are: gender, age group (18 to 24, 25 to 34, 35 to 44, 45 to 54, 55 to 64, 65 or more) and educational level (0 to 8, 9 to 11, 12 or more years of study). Poisson regression models were used to describe the crude and adjusted associations between sociodemographic variables and the frequency of high consumption of ultra-processed foods. All analyses were performed using the Stata software, version 16.1, always considering the sample weights of the survey that make the sociodemographic distribution of the Vigitel sample in each capital identical to that projected for 2019 based on census data.

The free and informed consent of the survey participants of the Vigitel system is obtained orally at the time of telephone contact with the interviewees and the project for conducting these surveys was approved by the National Commission of Research Ethics for Studies with Human Beings of the Ministry of Health (CAAE: 65610017.1.0000.0008).

## RESULTS

Table 1 describes the frequency of consumption on the day before the interview for each of the 13 selected subgroups of ultra-processed foods for the adult population of the 27 capitals as a whole. Those who reached the highest frequency of consumption were: margarine (42.6%); loaf, hot dog or hamburger bread (32.8%); soft drink (27.7%); sausage, mortadella or ham (26.5%); chocolate, ice cream, gelatin, *flan* or other industrialized dessert (25.6%); packaged salty snacks or crackers (23.9%); and sweet cookie, stuffed cookie or packaged cake (21.3%). The remaining subgroups of ultra-processed foods were consumed by less than 20% of respondents.

**Table 1:** Frequency (%) of consumption of selected groups of ultra-processed foods on the previous day. Adult population (≥ 18 years old) from the capitals of the 27 federative units in Brazil, 2019 (n = 52,443).

Ultra-processed food groups	% (95%CI)
Margarine	42.6 (41.7–43.6)
Loaf, hot dog or hamburger bread	32.8 (31.9–33.7)
Soft drink	27.7 (26.8–28.6)
Sausage, mortadella or ham	26.5 (25.6–27.4)
Chocolate, ice cream, gelatin, *flan* or other industrialized dessert	25.6 (24.8–26.5)
Packaged salty snacks or crackers	23.9 (23.1–24.7)
Sweet cookie, stuffed cookie or packaged cake	21.3 (20.5–22.1)
Mayonnaise, ketchup or mustard	16.9 (16.1–17.6)
Flavored yogurt	15.6 (14.9–16.3)
Fruit juice in box or can	15.0 (14.3–15.8)
Powdered soft drink	12.8 (12.0–13.5)
Chocolate drink	11.9 (11.2–12.6)
Instant noodles, instant powdered soup, frozen lasagna or other ready-to-eat frozen dish	6.6 (6.1–7.2)

95%CI: 95% confidence interval.

The distribution of the score for the consumption of ultra-processed foods (number of subgroups consumed on the day before the interview) varied between zero and 13, with evident asymmetry on the right and concentration of values between 1 and 4 (18.7%, 20.0%, 18.9%, 13.6%, respectively). The frequency of score zero was 10.6% and the frequency of score equal to five reached 8.6%, with the frequencies of higher scores being progressively lower (Figure).

**Figure:**
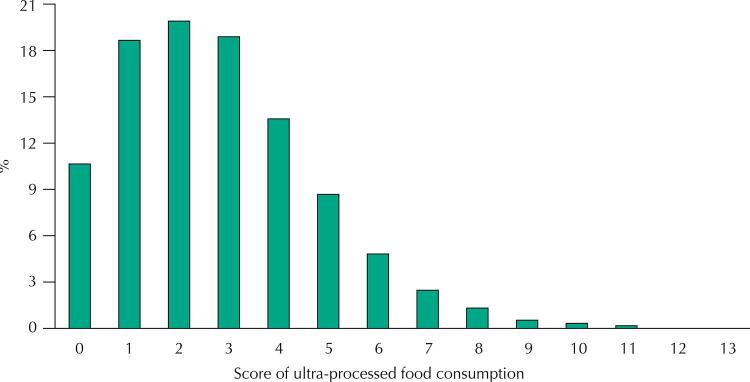
Distribution (%) of the population according to the ultra-processed food consumption scorea. Adult population (≥ 18 years old) of the capitals of the 27 federative units of Brazil, 2019 (n = 52,443).

^a^ Number of ultra-processed food subgroups (n = 13) consumed on the day before the interview.

The frequency of high consumption of ultra-processed foods – scores equal to or greater than five – was 18.2% (95% CI 17.4–19.0) in the adult population of the 27 capitals (Table 2). With or without adjustment for the other sociodemographic variables, this frequency was significantly lower in females and decreased linearly with age. In the crude analysis, the relationship between the frequency of high consumption of ultra-processed foods and schooling presented an inverted U-shape, increasing from the lower to the intermediate level and decreasing from the lower to the upper level. In the adjusted analysis, the frequency of high consumption was significantly lower at the higher level of education (12 or more years of study), with no significant differences between the lower level (less than 8 years of study) and the intermediate level (9 to 11 years of study) (Table 2). There were no significant interactions between sex and other sociodemographic variables (age and education) in their association with the high consumption of ultra-processed foods.

**Table 2:** Frequency of ultra-processed food consumption scores ≥ 5 according to sociodemographic variables. Adult population (≥ 18 years old) from the capitals of the 27 federative units in Brazil, 2019 (n = 52,443).

Variables	% (95%CI) of scores ≥ 5	
	Crude	Adjusted^a^
Sex		
Male	21.8 (20.5–23.2)	20.9 (19.6–22.2)
Female	15.1 (14.2–16.1)^b^	15.8 (14.8–16.7)^b^
Age (years)		
18–24	29.3 (26.9–31.6)	28.6 (26.1–31.1)
25–34	23.6 (21.5–25.8)	24.2 (21.9–26.5)
35–44	19.1 (17.4–20.9)	19.4 (17.6–21.1)
45–54	13.9 (12.3–15.5)	13.8 (12.2–15.3)
55–64	9.8 (8.4–11.1)	9.7 (8.4–11.0)
65 or more	8.0 (7.1–8.9)^c^	7.9 (7.0–8.9)^c^
Educational level (years)		
0–8	14.7 (13.2–16.2)	19.6 (17.4–21.8)
9–11	21.7 (20.3–23.0)^d^	19.7 (18.5–20.9)
12 or more	17.3 (16.0–18.6)^e.f^	15.7 (14.5–16.9)^e.f^
Total	18.2 (17.4–19.0)	-

95%CI: 95% confidence interval

^a^ Adjustment for other sociodemographic variables using Poisson regression.

^b^ p < 0.001.

^c^ p < 0.001 for linear trend.

^d^ p < 0.001 in comparison with category 0 to 8 years.

^e^ p < 0.001 in comparison with category 9 to 11 years.

^f^ p < 0.05 in comparison with category 0 to 8 years.

## DISCUSSION

Based on a new module of questions from the Vigitel system on the previous day’s consumption of 13 subgroups of ultra-processed foods, selected from those most consumed in Brazil, it was estimated that in 2019 nine out of 10 people in the adult Brazilian population of the capitals of the 27 federation units consumed at least one subgroup and one in five consumed five or more subgroups of ultra-processed foods. Margarine, loaves of bread and similar, soft drinks, sausages, industrialized desserts and salty snacks or sweet cookies were the subgroups most frequently consumed. Multiple regression analyses showed that the consumption of five or more subgroups of ultra-processed foods decreased linearly with age and was lower in women and for people with university education.

The inverse linear association between consumption of ultra-processed foods and age identified by our study in Brazil has been reported in other countries by studies that considered the daily intake of the set of ultra-processed foods^7,22^. This association could reflect either a cohort effect or a greater health concern among older people, or, more likely, both factors. In any case, the higher consumption of ultra-processed foods among young adults – one in four people between 18 and 34 years of age consumed five or more subgroups of ultra-processed foods on the previous day – justifies special attention to this group, as well as to children and adolescents, in public policies that aim to reduce the consumption of ultra-processed foods, such as the restriction on the advertising of these products.

The higher consumption of ultra-processed foods among men, identified in our study, coincides with that found in Canada^7^ and the United Kingdom^22^, but not in Chile^23^, where consumption was higher among women, and in the United States^25^, Mexico^24^ and Colombia^26^, where the consumption was similar for both sexes. Inverse associations between consumption of ultra-processed foods and socioeconomic status, such as those we found for schooling in our study, are described in Canada^7^ and the United States^25^, while direct associations are reported in Chile^23^, Mexico^24^ and Colombia^26^.

This inconsistency observed in the literature regarding associations with sex and socioeconomic indicators may be due to differences in the mechanisms that, in each country, relate these variables to the consumption of ultra-processed foods. These mechanisms may be studied in Brazil as soon as the database of the National Health Survey 2019^27^ becomes available, since this research collected both information on socioeconomic status and consumption of ultra-processed foods and on a wide range of variables potentially associated with these two conditions. In any case, the higher consumption of ultra-processed foods evidenced in our study among men and people with less education justifies the prioritization of these strata in public policies that aim to reduce the consumption of ultra-processed foods in Brazil.

The module of questions on the consumption of subgroups of ultra-processed foods, initially developed for the Vigitel system, has already been used in two national surveys of the IBGE: the National Health Survey of 2019^27^ and the National Survey of School Health of 2019-2020^28^. Thus, the estimates on the consumption of ultra-processed foods described in this article for the adult population of the 27 Brazilian capitals will be briefly known to the country’s adult population as a whole and to the adolescent school population.

Trends in the evolution of the consumption of ultra-processed foods in Brazil can be estimated when Vigitel has a historical series of the score for the consumption of ultra-processed foods, similar to what the system provides for other indicators. In 2018, the first year of application of the module of questions on the consumption of subgroups of ultra-processed foods in the system, the frequency of scores ≥ 5 was 17.8% (95% CI 17.0–18.6) and the most recent application of this module, in the 2020 wave, it shows a frequency of scores ≥ 5 of 18.5 (95% CI 17.3–19.7) (estimates calculated especially for this article).

It is worth mentioning that the score for the consumption of ultra-processed foods was validated in a study conducted in 2018 with a convenience sample of 150 adults selected from the clientele of two basic health units in the city of São Paulo. The validation study showed substantial agreement (PABAK index: 0.72) between fifths of the score for the consumption of ultra-processed foods (measured by a questionnaire identical to that used in Vigitel) and fifths of the dietary share of ultra-processed foods (% of total calories, measured by 24-hour dietary recall), both calculated based on the previous day’s food consumption^29^. A second study was carried out in 2019, with a convenience sample of 300 adults, to assess the performance of the score of ultra-processed food consumption adapted for self-filling, through the breakdown of some of the 13 subgroups of the instrument used in Vigitel, totaling 23 subgroups of ultra-processed foods. The data showed a similar agreement (PABAK index: 0.67) when comparing the fifths of the score to the fifths of the energy contribution from ultra-processed foods^30^.

A limitation of the Vigitel system is the inclusion of fixed telephone lines only in its sampling process, despite the fact that fixed telephone coverage is lower than that of mobile telephony in most Brazilian cities. For future studies, the Vigitel system should soon include in its sampling process the drawing of samples of cell phones in order to improve the estimates of all its indicators, especially in capitals where the coverage of the fixed telephone service is very low, as in the capitals of the North and Northeast regions^31^.

In conclusion, the results of the present study indicate that ultra-processed foods are consumed with high frequency in the adult Brazilian population of the 27 capitals of the federation and that belonging to the male gender, being younger and having less than university education are conditions that increase, independently, the consumption of those foods. Taking into account the evidence that demonstrates the harmful effect of the consumption of ultra-processed foods on the quality of the diet and on the risk of several chronic non-communicable diseases, public policies that reduce the consumption of these foods are justified, especially in the male population, in young people, and in the less educated strata. Monitoring the score of ultra-processed food consumption by the Vigitel system will be important to assess the success of these policies.
