# Supplementary material for: Consumption of ultra-processed foods and its association with sociodemographic factors in the adult population of the 27 Brazilian state capitals (2019)
Source: Rev Saude Publica. 2021 Jul 27;55:47. doi: 10.11606/s1518-8787.2021055002833 (PMC8317992; doi:10.11606/s1518-8787.2021055002833)
Supplement: Translation

# Translation: Consumo de alimentos ultraprocessados e associação com fatores sociodemográficos na população adulta das 27 capitais brasileiras (2019)

**Authors:** Caroline dos Santos Costa, Isabela Fleury Sattamini, Eurídice Martinez Steele, Maria Laura da Costa Louzada, Rafael Moreira Claro, Carlos Augusto Monteiro

**Affiliations:** I Brasil Universidade de São Paulo. Faculdade de Saúde Pública. Programa de Pós-Graduação em Nutrição em Saúde Pública. São Paulo, SP, Brasil; II Brasil Universidade de São Paulo. Núcleo de Pesquisas Epidemiológicas em Nutrição e Saúde. São Paulo, SP, Brasil; III Brasil Universidade de São Paulo. Faculdade de Saúde Pública. Departamento de Nutrição. São Paulo, SP, Brasil; IV Brasil Universidade Federal de Minas Gerais. Escola de Enfermagem. Departamento de Nutrição. Belo Horizonte, MG, Brasil

**Keywords:** Dieta, Adultos, Alimentos Ultraprocessados, Distribuição Sociodemográfica, Brasil

## Abstract

**OBJETIVO:**

Descrever a magnitude do consumo de alimentos ultraprocessados na população adulta (≥ 18 anos) das capitais das 27 unidades federativas do Brasil e sua associação com variáveis sociodemográficas.

**MÉTODOS:**

Os dados utilizados neste estudo provêm dos participantes (n = 52.443) da onda 2019 do inquérito anual do “Sistema nacional de vigilância de fatores de risco e proteção para doenças crônicas por inquérito telefônico” (Vigitel). O consumo de alimentos ultraprocessados foi descrito com base em escore correspondente à somatória de respostas positivas para questões sobre o consumo no dia anterior de treze subgrupos de alimentos ultraprocessados frequentemente consumidos no Brasil. Modelos de regressão de Poisson foram utilizados para descrever as associações bruta e ajustada do alto consumo de alimentos ultraprocessados (escores ≥ 5) com sexo, faixa etária e nível de escolaridade.

**RESULTADOS:**

A frequência de alto consumo de alimentos ultraprocessados foi 18,2% (IC95% 17,4–19,0). Com ou sem o ajuste para as demais variáveis sociodemográficas, essa frequência foi significativamente menor no sexo feminino e diminuiu linearmente com a idade. Na análise bruta, evidenciou-se aumento na frequência de alto consumo do nível inferior para o nível intermediário de escolaridade e diminuição desse consumo do nível intermediário para o superior. Na análise ajustada por sexo e idade, a frequência de alto consumo de alimentos ultraprocessados foi significativamente menor no nível superior de escolaridade (12 ou mais anos de estudo), não havendo diferenças entre os demais níveis.

**CONCLUSÃO:**

Alimentos ultraprocessados são consumidos com alta frequência na população brasileira adulta das 27 capitais da federação. Pertencer ao sexo masculino, ser mais jovem e ter escolaridade inferior à universitária são condições que aumentam, de forma independente, o consumo desses alimentos.

## INTRODUÇÃO

Alimentos ultraprocessados são formulações industriais de substâncias derivadas de alimentos que contêm pouco ou nenhum alimento inteiro e frequentemente são adicionadas de aromatizantes, corantes, emulsificantes e outros aditivos com função cosmética^1,2^. Séries temporais sobre a venda de alimentos ultraprocessados em 80 países indicam aumentos anuais substanciais no consumo global desses produtos, sobretudo em países de regiões economicamente menos desenvolvidas^3^.

No Brasil, a venda de alimentos ultraprocessados correspondeu a 448 kcal per capita/dia em 2014, valor 10,4% superior ao registrado em 2009^4^. Inquéritos nacionais de compras de alimentos mostram que a contribuição de alimentos ultraprocessados para o total das calorias adquiridas pelos domicílios brasileiros aumentou de 12,6% em 2002–2003 para 16,0% em 2008–2009 e para 18,4% em 2017–2018^5^. Em 2017–2018, alimentos ultraprocessados representaram cerca de 20% do total de calorias ingeridas por adolescentes e adultos no Brasil^5^.

Estudos em amostras representativas da população de vários países, entre eles o Brasil, mostram que o consumo de alimentos ultraprocessados está associado a dietas com maior densidade energética, com mais açúcar e gorduras não saudáveis e com menor teor de fibra, proteína, vitaminas e minerais e, portanto, a dietas que aumentam o risco de doenças crônicas não transmissíveis (DCNT)^6^. Revisões sistemáticas da literatura sobre estudos de coorte realizados com populações adultas do Brasil e de outros países evidenciam que o consumo de alimentos ultraprocessados está efetivamente associado à maior incidência de DCNT, incluindo obesidade, hipertensão arterial, diabetes, doenças cardiovasculares, câncer e depressão^12,13^. Três estudos de coorte^14^ identificaram associações entre consumo de alimentos ultraprocessados e mortalidade por todas as causas e um ensaio clinico randomizado *cross-over* mostrou que dietas ultraprocessadas determinam um aumento diário de cerca de 500 kcal no consumo alimentar e, em duas semanas, uma diferença acumulada de cerca de 2 kg no peso corporal^17^.

Em função das evidências que relacionam alimentos ultraprocessados à saúde e da tendência ascendente da participação desses alimentos na dieta brasileira, uma das recomendações centrais do guia alimentar do Ministério da Saúde do Brasil é evitar o consumo de alimentos ultraprocessados^18^.

Desde 2006, o Brasil conta com um sistema nacional de monitoramento anual de fatores de risco e proteção para doenças crônicas não transmissíveis. Esse sistema, denominado Vigitel (“Sistema nacional de vigilância de fatores de risco e proteção para doenças crônicas por inquérito telefônico”), foi desenvolvido e testado na capital do Estado de São Paulo entre 2003 e 2005^19,20^. O Vigitel opera por meio de entrevistas telefônicas realizadas em amostras probabilísticas da população adulta com telefone fixo residente nas capitais de todas as 27 unidades da federação. Utilizando pesos amostrais, o sistema fornece estimativas anuais da frequência e distribuição de fatores de risco ou proteção para doenças crônicas para a população adulta residente em cada capital e no conjunto das capitais^21^. Por exemplo, com base no sistema Vigitel, estima-se que, entre 2006 e 2019, a prevalência da obesidade na população adulta do conjunto das 27 capitais brasileiras aumentou de 11,8% para 20,3%, enquanto a prevalência de fumantes diminuiu de 15,7% para 9,8%.

Em 2019, o inquérito telefônico anual do sistema Vigitel incluiu um módulo de questões sobre o consumo de alimentos ultraprocessados. Com base nesse módulo, o presente estudo objetivou estimar o consumo de alimentos ultraprocessados na população adulta residente nas capitais das 27 unidades federativas do Brasil e investigar sua associação com variáveis sociodemográficas.

## MÉTODOS

Todos os dados utilizados neste estudo são provenientes da onda 2019 do inquérito transversal anual do sistema Vigitel^21^.

### Amostragem

Em 2019, o sistema Vigitel entrevistou 52.443 pessoas com 18 ou mais anos de idade residentes nas capitais das 27 unidades federativas do Brasil.

O processo de amostragem utilizado pelo Vigitel é descrito em detalhe nos relatórios anuais do sistema^21^. Resumidamente, com base em cadastros das operadoras de telefonia fixa, são sorteadas cinco mil linhas telefônicas fixas por capital por sorteio sistemático e estratificado por código de endereçamento postal (CEP). A seguir, essas linhas são agrupadas em réplicas de 200 linhas, cada réplica reproduzindo a mesma proporção de linhas por CEP do cadastro original. Linhas elegíveis são aquelas correspondentes a residências onde haja pelo menos um morador com 18 anos de idade ou mais. Para cada linha elegível, sorteia-se um dos moradores adultos e este é entrevistado. Ligações são feitas para as linhas contidas em cada réplica sucessivamente e até que se chegue a um total de aproximadamente duas mil entrevistas em cada capital.

### Coleta de Dados

As entrevistas do sistema Vigitel são realizadas ao longo dos doze meses do ano através de ligações telefônicas feitas por operadores treinados. O questionário do sistema contempla perguntas com breve enunciado e com categorias de resposta pré-codificadas sobre características sociodemográficas, consumo de tabaco e de bebidas alcoólicas, alimentação e atividade física, entre outras^21^. Em 2019, o questionário do Vigitel incluiu um módulo de questões sobre o consumo no dia anterior (sim ou não) de 13 subgrupos de alimentos ultraprocessados, selecionados dentre aqueles mais consumidos no Brasil segundo o inquérito nacional de consumo alimentar realizado como parte da Pesquisa de Orçamentos Familiares (POF) do IBGE de 2008–2009^6^. São eles: refrigerantes; suco de fruta em caixa, caixinha ou lata; refresco em pó; bebida achocolatada; iogurte com sabor; salgadinho de pacote (ou chips) ou biscoito/bolacha salgado; biscoito/bolacha doce, biscoito recheado ou bolinho de pacote; chocolate, sorvete, gelatina, *flan* ou outra sobremesa industrializada; salsicha, linguiça, mortadela ou presunto; pão de forma, de cachorro-quente ou de hambúrguer; maionese, ketchup ou mostarda; margarina; macarrão instantâneo, sopa de pacote, lasanha congelada ou outro prato pronto comprado congelado.

### Análise de Dados

Inicialmente, descrevemos para o conjunto da população adulta das 27 capitais a frequência de consumo no dia anterior à entrevista de cada um dos 13 subgrupos de alimentos ultraprocessados incluídos no questionário do sistema Vigitel. Em seguida, calculamos para cada indivíduo um escore de consumo de alimentos ultraprocessados que corresponde à somatória de respostas positivas para as questões sobre o consumo de cada um dos subgrupos de alimentos ultraprocessados, podendo variar, portanto, entre zero e 13. A distribuição desse escore foi apresentada na forma gráfica.

Estimativas da frequência de alto consumo de alimentos ultraprocessados (desfecho, definido por escores ≥ 5) foram apresentadas para o conjunto da população adulta das 27 capitais e segundo variáveis sociodemográficas (exposições), sendo elas: sexo, faixa etária (18 a 24, 25 a 34, 35 a 44, 45 a 54, 55 a 64, 65 ou mais) e nível de escolaridade (0 a 8, 9 a 11, 12 ou mais anos de estudo). Modelos de regressão de Poisson foram utilizados para descrever as associações bruta e ajustada entre as variáveis sociodemográficas e a frequência do alto consumo de alimentos ultraprocessados. Todas as análises foram realizadas no software Stata, versão 16.1, sempre considerando os pesos amostrais da pesquisa que tornam a distribuição sociodemográfica da amostra Vigitel em cada capital idêntica à projetada para 2019 com base em dados censitários.

O consentimento livre e esclarecido dos participantes do inquérito do sistema Vigitel é obtido oralmente no momento do contato telefônico com os entrevistados e o projeto para a realização desses inquéritos foi aprovado pela Comissão Nacional de Ética em Pesquisa para Seres Humanos do Ministério da Saúde (CAAE: 65610017.1.0000.0008).

## RESULTADOS

A Tabela 1 descreve a frequência de consumo no dia anterior à entrevista de cada um dos 13 subgrupos selecionados de alimentos ultraprocessados para o conjunto da população adulta das 27 capitais. Os que alcançaram maiores frequências de consumo foram: margarina (42,6%); pão de forma, de cachorro-quente ou de hambúrguer (32,8%); refrigerante (27,7%); salsicha, linguiça, mortadela ou presunto (26,5%); chocolate, sorvete, gelatina, *flan* ou outra sobremesa industrializada (25,6%); salgadinho de pacote ou biscoito/bolacha salgado (23,9%); e biscoito/bolacha doce, biscoito recheado ou bolinho de pacote (21,3%). Os demais subgrupos de alimentos ultraprocessados foram consumidos por menos de 20% dos entrevistados.

**Tabela 1:** Frequência (%) de consumo no dia anterior de grupos selecionados de alimentos ultraprocessados. População adulta (≥ 18 anos) das capitais das 27 unidades federativas do Brasil, 2019 (n = 52.443).

Grupos de alimentos ultraprocessados	% (IC95%)
Margarina	42,6 (41,7–43,6)
Pão de forma, de cachorro-quente ou de hambúrguer	32,8 (31,9–33,7)
Refrigerante	27,7 (26,8–28,6)
Salsicha, linguiça, mortadela ou presunto	26,5 (25,6–27,4)
Chocolate, sorvete, gelatina, *flan* ou outra sobremesa industrializada	25,6 (24,8–26,5)
Salgadinho de pacote (ou chips) ou biscoito/bolacha salgado	23,9 (23,1–24,7)
Biscoito/bolacha doce, biscoito recheado ou bolinho de pacote	21,3 (20,5–22,1)
Maionese, ketchup ou mostarda	16,9 (16,1–17,6)
Iogurte com sabor	15,6 (14,9–16,3)
Suco de fruta em caixa, caixinha ou lata	15,0 (14,3–15,8)
Refresco em pó	12,8 (12,0–13,5)
Bebida achocolatada	11,9 (11,2–12,6)
Macarrão instantâneo, sopa de pacote, lasanha congelada ou outro prato pronto comprado congelado	6,6 (6,1–7,2)

IC95%: intervalo de confiança de 95%.

A distribuição do escore de consumo de alimentos ultraprocessados (número de subgrupos consumidos no dia anterior à entrevista) variou entre zero e 13, com evidente assimetria à direita e concentração de valores entre 1 e 4 (18,7%, 20,0%, 18,9%, 13,6%, respectivamente). A frequência de escore zero foi de 10,6% e a frequência de escore igual a cinco alcançou 8,6%, sendo progressivamente menores as frequências de escores superiores (Figura).

**Figura:** Distribuição (%) da população segundo escore de consumo de alimentos ultraprocessadosa. População adulta (≥ 18 anos) das capitais das 27 unidades federativas do Brasil, 2019 (n = 52.443).

^a^ Número de subgrupos de alimento ultraprocessados (n = 13) consumidos no dia anterior à entrevista.

A frequência de alto consumo de alimentos ultraprocessados – escores iguais ou superiores a cinco – foi de 18,2% (IC95% 17,4–19,0) na população adulta das 27 capitais (Tabela 2). Com ou sem ajuste para as demais variáveis sociodemográficas, essa frequência foi significativamente menor no sexo feminino e diminuiu linearmente com a idade. Na análise bruta, a relação da frequência de alto consumo de alimentos ultraprocessados com a escolaridade apresentou formato de U-invertido, aumentando do nível inferior para o intermediário e diminuindo deste para o superior. Na análise ajustada, a frequência do alto consumo foi significativamente menor no nível superior de escolaridade (12 ou mais anos de estudo), não havendo diferenças significativas entre o nível inferior (menos de 8 anos de estudo) e o nível intermediário (9 a 11 anos de estudo) (Tabela 2). Não foram observadas interações significativas entre sexo e as demais variáveis sociodemográficas (idade e escolaridade) em sua associação com o alto consumo de alimentos ultraprocessados.

**Tabela 2:** Frequência de escores de consumo de alimentos ultraprocessados ≥ 5 segundo variáveis sociodemográficas. População adulta (≥ 18 anos) das capitais das 27 unidades federativas do Brasil, 2018 (n = 52.443).

Variáveis	% (IC95%) de escores ≥ 5	
	Bruta	Ajustada^a^
Sexo		
Masculino	21,8 (20,5–23,2)	20,9 (19,6–22,2)
Feminino	15,1 (14,2–16,1)^b^	15,8 (14,8–16,7)^b^
Idade (anos)		
18 a 24	29,3 (26,9–31,6)	28,6 (26,1–31,1)
25 a 34	23,6 (21,5–25,8)	24,2 (21,9–26,5)
35 a 44	19,1 (17,4–20,9)	19,4 (17,6–21,1)
45 a 54	13,9 (12,3–15,5)	13,8 (12,2–15,3)
55 a 64	9,8 (8,4–11,1)	9,7 (8,4–11,0)
65 e mais	8,0 (7,1–8,9)^c^	7,9 (7,0–8,9)^c^
Escolaridade (anos)		
0 a 8	14,7 (13,2–16,2)	19,6 (17,4–21,8)
9 a 11	21,7 (20,3–23,0)^d^	19,7 (18,5–20,9)
12 e mais	17,3 (16,0–18,6)^e,f^	15,7 (14,5–16,9)^e,f^
Total	18,2 (17,4–19,0)	-

IC95%: intervalo de confiança de 95%.

^a^Ajuste para as demais variáveis sociodemográficas por regressão de Poisson.

^b^ p < 0,001.

^c^ p < 0,001 para tendência linear.

^d^ p < 0,001 na comparação com a categoria 0 a 8 anos.

^e^ p < 0,001 na comparação com a categoria 9 a 11 anos.

^f^ p < 0,05 na comparação com a categoria 0 a 8 anos.

## DISCUSSÃO

Com base em um novo módulo de questões do sistema Vigitel sobre o consumo no dia anterior de 13 subgrupos de alimentos ultraprocessados, selecionados dentre aqueles mais consumidos no Brasil, estimou-se que, em 2019, nove em cada 10 pessoas da população brasileira adulta das capitais das 27 unidades da federação consumiram pelo menos um subgrupo e uma em cinco consumiram cinco ou mais subgrupos de alimentos ultraprocessados. Margarina, pães de forma e assemelhados, refrigerantes, embutidos, sobremesas industrializadas e biscoitos salgados ou doces foram os subgrupos consumidos com mais frequência. Análises de regressão múltipla evidenciaram que o consumo de cinco ou mais subgrupos de alimentos ultraprocessados diminuiu linearmente com a idade e foi menor no sexo feminino e para as pessoas com escolaridade universitária.

A associação linear inversa entre consumo de alimentos ultraprocessados e idade identificada por nosso estudo no Brasil vem sendo relatada em outros países por estudos que consideraram a ingestão diária do conjunto de alimentos ultraprocessados^7,22^. Essa associação poderia refletir tanto um efeito de coorte ou uma maior preocupação com a saúde entre as pessoas mais velhas, ou, mais provavelmente, ambos os fatores. De qualquer forma, o maior consumo de alimentos ultraprocessados entre os adultos jovens – uma em cada quatro pessoas entre 18 e 34 anos de idade consumiu cinco ou mais subgrupos de alimentos ultraprocessados no dia anterior – justifica uma atenção especial a esse grupo, bem como às crianças e adolescentes, nas políticas públicas que visam reduzir o consumo de alimentos ultraprocessados, como a restrição à publicidade desses produtos.

O maior consumo de alimentos ultraprocessados entre homens, identificado em nosso estudo, coincide com o encontrado no Canadá^7^ e Reino Unido^22^, mas não no Chile^23^, onde o consumo foi maior entre mulheres, e nos Estados Unidos^25^, no México^24^ e na Colômbia^26^, onde o consumo foi similar nos dois sexos. Associações inversas entre consumo de alimentos ultraprocessados e nível socioeconômico, como as que encontramos para escolaridade em nosso estudo, são descritas no Canadá^7^ e Estados Unidos^25^, enquanto associações diretas são relatadas no Chile^23^, México^24^ e Colômbia^26^.

Essa inconstância observada na literatura quanto às associações com o sexo e com indicadores socioeconômicos pode se dever a diferenças nos mecanismos que, em cada país, relacionam essas variáveis ao consumo de alimentos ultraprocessados. Esses mecanismos poderão ser estudados no Brasil assim que a base de dados da Pesquisa Nacional de Saúde de 2019^27^ estiver disponível, uma vez que essa pesquisa coletou tanto informações sobre nível socioeconômico e consumo de alimentos ultraprocessados quanto sobre uma ampla gama de variáveis potencialmente associadas a essas duas condições. De qualquer forma, o maior consumo de alimentos ultraprocessados evidenciado em nosso estudo entre homens e pessoas com menor escolaridade justifica a priorização desses estratos nas políticas públicas que visam reduzir o consumo de alimentos ultraprocessados no Brasil.

O módulo de questões sobre o consumo de subgrupos de alimentos ultraprocessados, desenvolvido inicialmente para o sistema Vigitel, já foi empregado em dois inquéritos nacionais do IBGE: a Pesquisa Nacional de Saúde de 2019^27^ e a Pesquisa Nacional de Saúde do Escolar de 2019–2020^28^. Assim, as estimativas sobre o consumo de alimentos ultraprocessados descritas neste artigo para a população adulta das 27 capitais brasileiras serão brevemente conhecidas para o conjunto da população adulta do país e para a população escolar de adolescentes.

Tendências da evolução do consumo de alimentos ultraprocessados no Brasil poderão ser estimadas quando o Vigitel dispuser de uma série histórica do escore de consumo de alimentos ultraprocessados, a exemplo do que o sistema proporciona para outros indicadores. Em 2018, primeiro ano de aplicação do módulo de questões sobre o consumo de subgrupos de alimentos ultraprocessados no sistema, a frequência de escores ≥ 5 foi de 17,8% (IC95% 17,0–18,6) e a aplicação mais recente desse módulo, na onda 2020, mostra uma frequência de escores ≥ 5 de 18,5 (IC95% 17,3–19,7) (estimativas calculadas especialmente para este artigo).

Cabe ressaltar que o escore de consumo de alimentos ultraprocessados foi validado em estudo realizado em 2018 com uma amostra de conveniência de 150 adultos selecionados da clientela de duas unidades básicas de saúde do município de São Paulo. O estudo de validação mostrou concordância substancial (índice PABAK: 0,72) entre quintos do escore de consumo de alimentos ultraprocessados (aferido por questionário idêntico ao utilizado no Vigitel) e quintos da contribuição de alimentos ultraprocessados para a ingestão diária total de energia (aferido por recordatório alimentar de 24 horas), ambos calculados com base no consumo alimentar do dia anterior^29^. Um segundo estudo foi realizado em 2019, com uma amostra de conveniência de 300 adultos, para avaliar a performance do escore de consumo de alimentos ultraprocessados adaptado para autopreenchimento, mediante o desdobramento de alguns dos 13 subgrupos do instrumento utilizado no Vigitel, totalizando 23 subgrupos de alimentos ultraprocessados. Os dados evidenciaram uma concordância similar (índice PABAK: 0,67) ao serem comparados os quintos do escore aos quintos da contribuição energética proveniente de alimentos ultraprocessados^30^.

Uma limitação do sistema Vigitel é a inclusão apenas de linhas telefônicas fixas no seu processo de amostragem, apesar de a cobertura de telefonia fixa ser menor que a da telefonia móvel em grande parte das cidades brasileiras. Para futuros estudos, o sistema Vigitel deve proximamente incluir no seu processo de amostragem o sorteio de amostras de telefones celulares visando melhorar as estimativas de todos os seus indicadores, sobretudo em capitais onde a cobertura do serviço de telefonia fixa é muito baixa, como nas capitais das regiões Norte e Nordeste^31^.

Em conclusão, os resultados do presente estudo indicam que alimentos ultraprocessados são consumidos com alta frequência na população brasileira adulta das 27 capitais da federação e que pertencer ao sexo masculino, ser mais jovem e ter escolaridade inferior à universitária são condições que aumentam, de forma independente, o consumo daqueles alimentos. Tendo em conta as evidências que demonstram o efeito danoso do consumo de alimentos ultraprocessados sobre a qualidade da dieta e sobre o risco de várias doenças crônicas não transmissíveis, justificam-se políticas públicas que reduzam o consumo desses alimentos, sobretudo na população masculina, em jovens e nos estratos de menor escolaridade. O monitoramento do escore de consumo de alimentos ultraprocessados pelo sistema Vigitel será importante para avaliar o sucesso dessas políticas.
